# The Enteric Nervous System as a Mediator of Microbiota‐Gut‐Brain Interactions in Parkinson's Disease

**DOI:** 10.1111/jnc.70339

**Published:** 2026-01-04

**Authors:** Luisa Valdetaro, Maria Carolina Ricciardi, Patricia Pereira Almeida, Milena Barcza Stockler‐Pinto, Ana Lucia Tavares‐Gomes

**Affiliations:** ^1^ Department of Physiology and Pharmacology, Biomedical Institute Universidade Federal Fluminense Niterói Brazil; ^2^ Neuroglial Interaction Lab, Neuroscience Program Universidade Federal Fluminense Niterói Brazil; ^3^ Pathology Post Graduate Program Universidade Federal Fluminense Niterói Brazil; ^4^ Nutrition Sciences Post Graduate Program Universidade Federal Fluminense Niterói Brazil; ^5^ Cardiovascular Sciences Post Graduate Program Universidade Federal Fluminense Niterói Brazil; ^6^ Neuroglial Interaction Lab, Neurobiology Department, Biology Institute Universidade Federal Fluminense Niterói Brazil

**Keywords:** enteric nervous system, gut microbiota, neuroinflammation, Parkinson's disease, short‐chain fatty acids

## Abstract

Parkinson's disease (PD) is a multifactorial neurodegenerative disorder in which gastrointestinal dysfunction is highly prevalent and often precedes motor symptoms. Although research on gut microbiota alterations in PD has expanded rapidly, inconsistent findings and the absence of a reproducible microbial signature reveal the limitations of a microbiota‐centered view. The enteric nervous system (ENS), the intrinsic neural network of the gut, has been comparatively overlooked and remains underexplored, yet mounting evidence indicates that it undergoes profound alterations in PD. Pathological changes in enteric neurons and glial cells, including α‐synuclein accumulation, disrupted neurotransmission, impaired epithelial barrier regulation, and neuroinflammation, not only contribute to gastrointestinal dysfunction but may also drive disease propagation along the gut–brain axis. In parallel, PD‐related dysbiosis alters microbial metabolites and immune signaling, disrupting ENS physiology. This review reframes PD gut pathology by emphasizing the ENS as a central mediator of microbiota–brain communication. We highlight potential key pathways underlying this crosstalk, including short‐chain fatty acids (SCFAs), Toll‐like receptor (TLR) signaling, and serotonergic circuits, which normally sustain ENS function but, in the context of PD, contribute to barrier impairment, neuroinflammation, and neuronal alterations. By integrating evidence from human studies and experimental models, we argue that investigating ENS–microbiota interactions provides a more comprehensive perspective on PD pathophysiology and may guide the identification of novel biomarkers and therapeutic approaches capable of addressing both gastrointestinal and neurological manifestations of the disease.

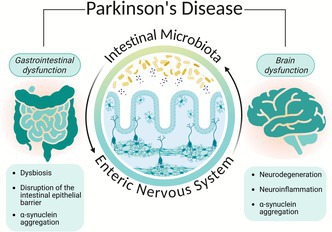

## Graphical abstract

Parkinson's disease (PD) is a neurodegenerative disorder that is increasingly recognized as a gut‐brain disorder, involving gut dysfunction before the onset of motor symptoms. While many studies have focused on changes in gut microbiota, inconsistent results highlight the limitations of viewing PD through a microbiota‐only lens. This review brings forward the underexplored role of the enteric nervous system (ENS), the gut's intrinsic neural network, as a central mediator of microbiota‐gut‐brain communication. By integrating evidence on microbial metabolites and signaling pathways, we discuss how ENS‐microbiota interactions may drive both gastrointestinal and brain pathology, offering new avenues for biomarkers and therapeutic strategies.

**Abbreviations**
5‐HT4R: serotonin 4 receptor
6‐OHDA: 6‐hydroxydopamine
AHR: aryl hydrocarbon receptor
ChAT: choline acetyltransferase
CNS: central nervous system
DHA: docosahexaenoic acid
DNs: dopaminergic neurons DMV, dorsal motor nucleus of the vagus
ENS: enteric nervous system
FMT: fecal microbiota transplantation
GDNF: glial cell line–derived neurotrophic factor
GF: germ‐free
GFAP: glial fibrillary acidic protein
GLP‐1: glucagon‐like peptide 1
GPR41: G‐protein coupled receptor 41
HDAC: histone deacetylase
IPANs: intrinsic primary afferent neurons
LPS: lipopolysaccharide
NaB: sodium butyrate
nNOS: Neuronal Nitric Oxide Synthase
PD: Parkinson's disease
PYY: peptide YY
ROS: reactive oxygen species
S100B: S100 calcium‐binding protein B
SCFAs: short‐chain fatty acids
TH: tyrosine hydroxylase
TLR: Toll‐like receptor
TLR2: Toll‐like receptor 2
TLR4: Toll‐like receptor 4
TNF‐α: tumor necrosis factor alpha
VIP: vasoactive intestinal peptide

## 1 Introduction

Parkinson's disease (PD) is a neurodegenerative disorder traditionally defined by its motor symptoms. However, it has become increasingly evident that PD is not confined to the central nervous system (CNS). Patients frequently experience a broad spectrum of non‐motor manifestations, including sleep disturbances, olfactory deficits, cardiovascular abnormalities, and, most prominently, gastrointestinal dysfunction (Schapira et al. 2017). Gastrointestinal symptoms such as constipation, delayed gastric emptying, and dysphagia affect more than 80% of individuals with PD and often precede motor signs by years or even decades (Warnecke et al. 2022). These symptoms not only diminish quality of life but also provide valuable insight into early disease processes that extend beyond the brain. Furthermore, pathological findings, such as the presence of α‐synuclein protein aggregates, highlight the gastrointestinal tract as a potential contributor to disease initiation and progression, suggesting that the gut plays a central role in PD (Warnecke et al. 2022).

Over the past decade, the gut microbiota has been the focus of intense investigation, with numerous studies reporting compositional and functional alterations in patients with PD. These observations have generated considerable interest in the possibility of a PD‐specific microbial “signature” and have driven efforts to elucidate how microbial metabolites and immune modulation might influence neurodegeneration. Despite these advances, however, the microbiota‐centered perspective faces significant challenges. Findings across studies have been inconsistent, and no robust or reproducible microbial signature has yet emerged (Santos et al. 2025). Moreover, attempts to manipulate the microbiota therapeutically have yielded limited and variable outcomes, underscoring the complexity of host–microbe interactions and suggesting that a narrow focus on the microbiota alone may not capture the full spectrum of PD pathophysiology.

Within this broader framework, the enteric nervous system (ENS) has received comparatively little attention, despite being the intrinsic neural network that regulates gut function and communicates bidirectionally with both the microbiota and the CNS. Anatomically, the ENS is organized into two major plexuses—the myenteric plexus, located between the longitudinal and circular muscle layers, and the submucosal plexus, situated within the submucosa—and comprises diverse neuronal subtypes and enteric glial cells. Together, these interconnected neuroglial networks autonomously coordinate motility, secretion, epithelial barrier regulation, and local blood flow, while engaging in close neuroimmune interactions within the intestinal wall. Evidence indicates that the ENS is profoundly altered in PD: enteric neurons and glial cells exhibit pathological changes, including α‐synuclein protein accumulation, disrupted neurochemical signaling, impaired barrier regulation, and neuroinflammation (Montanari et al. 2023). These alterations not only contribute to gastrointestinal dysfunction but may also facilitate disease propagation along the gut–brain axis (Warnecke et al. 2022). Importantly, the ENS serves as a physiological interface through which the gut microbiota exerts many of its effects on host physiology, positioning it as a critical mediator rather than a passive target.

We argue that advancing the understanding of PD requires a shift in focus from the microbiota in isolation to the interactions among the microbiota, the ENS, and the host. The ENS integrates microbial, immune, and neural signals, shaping both local gut physiology and systemic brain–gut communication. Neglecting this dimension risks oversimplifying the pathogenesis of PD and overlooking therapeutic opportunities. In this review, we examine current knowledge of ENS alterations in PD and critically evaluate their intersection with changes in the gut microbiota. We propose that a comprehensive exploration of this interplay is essential to unraveling the role of the gut–brain axis in PD and to identifying strategies capable of addressing both motor and non‐motor aspects of the disease.

## 2 ENS in PD: A Key Mediator of Gastrointestinal Dysfunction and Gut‐Brain Crosstalk

Over the past four decades, it has become increasingly evident that PD is not confined to the CNS but also affects the peripheral nervous system, including the gastrointestinal tract. As previously noted, gastrointestinal dysfunction in PD not only compromises patients' quality of life but may also participate in the earliest stages of the disorder (Warnecke et al. 2022). At present, three major lines of inquiry guide gastrointestinal‐related PD research: (1) elucidating gastrointestinal pathology to improve symptom management; (2) identifying metabolite biomarkers, given the accessibility of the gut; and (3) investigating the contribution of the gut to early pathogenic mechanisms of PD (Shannon and vanden Berghe 2018).

In this context, the ENS, the division of the autonomic nervous system located in the gut, has become a key focus in PD research. Numerous alterations have been reported in the ENS of both PD patients and animal models, including changes that precede the onset of motor symptoms. Biochemical and functional abnormalities in enteric neurons and enteric glial cells have been directly linked to gastrointestinal dysmotility, increased intestinal epithelial barrier permeability, neuroinflammation, and disease progression in PD (Montalbán‐Rodríguez et al. 2024; Montanari et al. 2023; Shannon and vanden Berghe 2018). In this section, we provide an overview of the main ENS alterations reported in PD.

The first studies connecting the ENS to PD were published in the 1980s, when Lewy neurites were identified in enteric neurons of PD patients (Wakabayashi 1989; Wakabayashi et al. 1990). Since these early reports, numerous studies have described Lewy neurites in enteric neurons from PD gastrointestinal biopsies and animal models; however, their exact role in PD pathophysiology remains unclear. The Braak hypothesis, proposed in 2003, suggests that the ENS and the olfactory bulb serve as initial sites of α‐synuclein protein aggregation, the main component of Lewy bodies and neurites, with subsequent propagation of pathology to the CNS via the vagus nerve (Braak et al. 2003). Although α‐synuclein transport between neurons has been demonstrated and gut‐to‐brain spread of α‐synuclein pathology has been documented in preclinical models, the underlying mechanisms and their relevance in human PD remain elusive (Bieri et al. 2018; Chen and Mor 2023; Uemura et al. 2021).

While Lewy bodies in the CNS are associated with neurodegeneration, evidence for enteric neuronal loss in PD is inconclusive, and no consensus exists on whether the ENS undergoes a true neurodegenerative process (O'Day et al. 2022). Age‐related enteric neuronal loss further complicates the distinction between PD‐specific changes and normal aging (Cirillo et al. 2013; Saffrey 2013). Moreover, most patient biopsy samples examine only the submucosal plexus, leaving the myenteric plexus, where most enteric neurons reside, largely unexplored. Lebouvier et al. reported a modest reduction in neurofilament M‐positive submucosal neurons in the colon, whereas other studies found no differences in duodenal and colonic neuron counts in PD subjects (Corbillé et al. 2014; Desmet et al. 2017; Lebouvier et al. 2008). Although full‐thickness gut analysis is feasible in rodent models, variability among PD models hinders definitive conclusions regarding enteric neurodegeneration (Han et al. 2025; Jiang et al. 2023; Palanisamy et al. 2022).

Conversely, multiple studies have documented changes in the chemical profile of the ENS that correlate with PD‐related gastrointestinal symptoms. Schaffernicht et al. showed that in a rotenone‐based PD animal model, excitatory cholinergic input in the ENS is reduced, leading to diminished gastrointestinal contractility and constipation before motor symptoms emerge (Schaffernicht et al. 2021). In patients with PD and chronic constipation, submucosal colonic neurons downregulate vasoactive intestinal peptide (VIP) without neuronal loss, suggesting that constipation may arise from dysfunction rather than depletion of VIP‐ergic secretomotor neurons (Chalazonitis and Rao 2018; Giancola et al. 2017). Altered dopaminergic and nitrergic signaling has also been reported and linked to PD symptoms (Anderson et al. 2007; Blandini et al. 2009; Coletto et al. 2021). A comprehensive characterization of the specific enteric neuronal subpopulations involved, and whether they undergo cell death or functional and phenotypic shifts, remains to be achieved.

Enteric glial cells, which are essential for epithelial integrity, neurotransmission, and immune regulation, have increasingly been recognized as key contributors to PD‐related gut dysfunction (Thomasi et al. 2023). In the gut, glial cells represent the main ENS component mediating immune–nervous system crosstalk, thereby contributing to neuroinflammation—a hallmark of many neurodegenerative diseases (Seguella and Gulbransen 2021). This process, characterized by persistent immune activation and subsequent neuronal damage, is considered a crucial pathological mechanism where the ENS plays a central role. Multiple studies have documented a reactive glial state in PD, marked by increased glial fibrillary acidic protein (GFAP) expression and changes in glial morphology and localization, which are linked to dysmotility, tissue damage, heightened epithelial barrier permeability, and dysbiosis (Emmi et al. 2023; Thomasi et al. 2023; Thomasi et al. 2024; Thomasi et al. 2022). These features collectively contribute to gut dysfunction in PD, though the specific relationships between them, such as the connection between glial activation and dysbiosis, need further research. The pathophysiological ENS changes discussed here, particularly those occurring in the early stages of PD, have significant clinical implications because gut tissue is accessible through endoscopy and colonoscopy. Indeed, intensive research on the ENS aims to establish an early diagnostic biomarker for PD. Among potential ENS biomarkers, Lewy pathology in enteric neurons is notable, as it can be detected in colonic biopsies up to 20 years before the onset of motor symptoms. α‐Synuclein accumulation appears to follow a rostrocaudal gradient, with temporal progression but a static spatial distribution over time (Shin et al. 2024). Glial markers such as GFAP have also been proposed as potential biomarkers, given that neuroinflammation is a hallmark of early gastrointestinal dysfunction in PD (Clairembault et al. 2014; Devos et al. 2013). However, the feasibility of using colonic biopsies for early PD diagnosis still faces many challenges. Current studies present several limitations, including the lack of a standardized comprehensive protocol, uncertainty regarding the most suitable gut region for sampling, sensitivity issues, and heterogeneity across populations (Pouclet et al. 2012; Schröder et al. 2025).

ENS involvement in PD has been extensively explored, yet the exact mechanisms underlying gastrointestinal symptoms and the spread of α‐synuclein pathology to the CNS remain unresolved. Methodological heterogeneity across studies complicates the interpretation of the ENS's role in PD. A review by Shannon and Van der Berghe highlights several outstanding questions, including how the ENS interacts with the gut microbiota, another critical player in PD pathophysiology, as discussed above (Shannon and vanden Berghe 2018). Despite extensive research on both the ENS and the microbiota, the ways in which their interaction contributes to PD development are still poorly understood.

## 3 Microbiota and PD: From Association to Mechanisms

Beyond the ENS, the gut microbiota has also emerged as a critical factor in PD pathophysiology. The intestinal microbial composition is now a central focus in studies investigating the disease. Over the past decade, a consistent association has been established between PD symptomatology and dysbiotic gut microbiota (del Rey et al. 2018; Qian et al. 2018). Clinical investigations across diverse populations worldwide (Nuzum et al. 2020; Mehanna et al. 2023; Proano et al. 2023; Zhang, Tang, and Guo 2023) have linked alterations in bacterial phyla, families, and genera to the multiple phenotypes of the disease, as well as onset time, duration, and disease state (Cilia et al. 2021; Hill‐Burns et al. 2017; Barichella et al. 2019). Compared with healthy individuals, patients with PD show an increased relative abundance of bacteria associated with inflammatory processes, along with a decrease in bacteria that support intestinal and systemic homeostasis (Ran et al. 2025). However, defining a specific gut microbial signature for PD remains challenging. A recent study reported elevated levels of *Streptococcus mutans* and its metabolite imidazole propionate in patients, identifying them as drivers of PD‐related features in mice, such as nigrostriatal dopaminergic degeneration and motor impairment. Nonetheless, further investigation is required to clarify how elevated levels of imidazole propionate contribute to PD pathophysiology (Park et al. 2025). The difficulty in defining a PD‐associated microbial signature stems from numerous individual and methodological limitations, including ethnic differences, sex‐based variation, dietary diversity, comorbidities, small sample sizes, varying analytical methods, a lack of longitudinal data, and insufficient exploration of bacterial interactions (Heravi et al. 2023).

**FIGURE 1:**
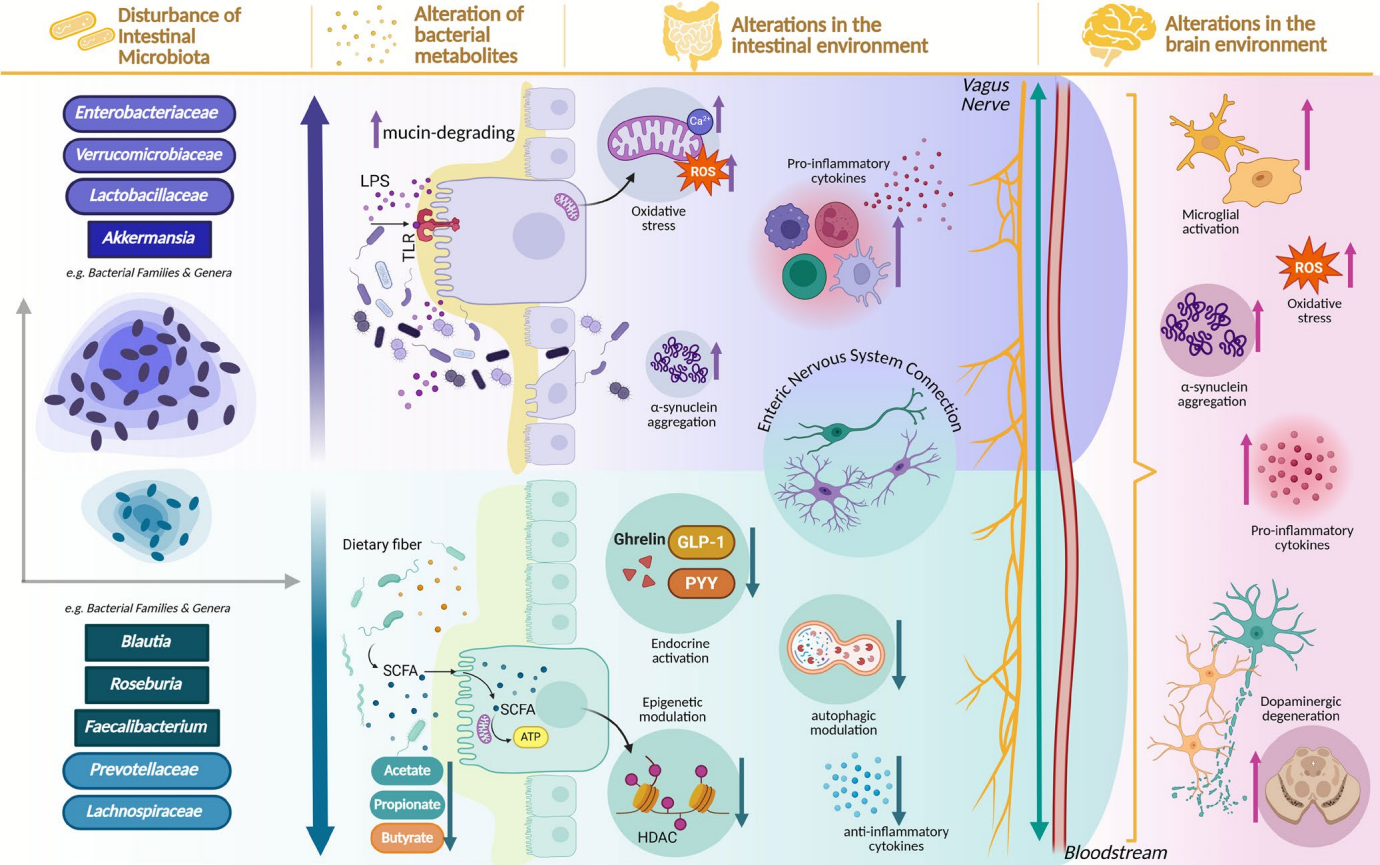
Schematic representation of microbiota‐driven modulation of the gut–brain axis in Parkinson's disease. Intestinal dysbiosis, characterized by an imbalance in the bacterial ecosystem, alters key metabolites such as lipopolysaccharide (LPS) and short‐chain fatty acids (SCFAs). Expansion of *Akkermansia muciniphila* , for example, promotes mucin degradation, weakening the luminal barrier and increasing epithelial permeability, which facilitates LPS translocation and amplifies intestinal inflammation, oxidative stress, and α‐synuclein aggregation. In parallel, the decrease of SCFA‐producing taxa reduces endocrine signaling [glucagon‐like peptide 1 (GLP‐1), ghrelin, peptide Y (PYY)], impairs autophagic and epigenetic regulation, and diminishes anti‐inflammatory cytokine production. These microbial and metabolic disturbances propagate via the enteric nervous system, vagus nerve, and bloodstream, ultimately driving microglial activation, oxidative stress, pro‐inflammatory cytokine release, α‐synuclein aggregation, and dopaminergic neurodegeneration in the brain. Created with http://biorender.com.

More than the fluctuating presence of specific microbial taxa, the shift in metabolic products and bioactive molecules derived from intestinal bacteria provides the most meaningful insight into PD (Ran et al. 2025) (Figure 1). In line with this perspective, several studies have consistently reported increased levels of *Enterobacteriaceae* and *Akkermansia*, taxa strongly linked to pro‐inflammatory and bioactive molecular changes, in fecal samples from patients with severe postural instability, gait difficulties, and exacerbated non‐motor symptoms (Scheperjans et al. 2015; Heintz‐Buschart et al. 2018; Zhang, Tang, and Guo 2023). Notably, repeated oral exposure to curli‐producing bacteria led to increased neuronal α‐syn deposition in both the gut and brain of aged rats (Cheng et al. 2023; Kustrimovic et al. 2024). In addition to this, the elevated abundance of these Gram‐negative, lipopolysaccharide (LPS)‐producing bacteria contributes to both intestinal and systemic inflammation and oxidative stress (Engler et al. 2009; Scheperjans et al. 2015) (Figure 1). For instance, *Akkermansia muciniphila* has been shown to enhance mitochondrial Ca^2+^ uptake in enterocyte cultures, resulting in mitochondrial dysfunction, increased reactive oxygen species (ROS) production, and pathological α‐synuclein aggregation in vitro (Amorim Neto et al. 2022). This epithelial injury aligns with the increased intestinal epithelial permeability observed in colonic tissue samples from PD patients (Clairembault et al. 2015). Compelling evidence suggest that under such conditions, LPS may translocate into intestinal tissue and interact with the complex enteric neuroglial network via Toll‐like receptor 4 (TLR4), which is abundantly expressed in the ENS of the distal colon in murine models and in the human ileum (Barajon et al. 2009; Boller and Felix 2009). Beyond local effects, LPS can enter the bloodstream, cross the compromised blood–brain barrier, and activate TLR4 on microglial cells (Clairembault et al. 2015; Perez‐Pardo et al. 2019; Blasco et al. 2020). It is widely recognized this activation induces the production of pro‐inflammatory cytokines and oxidative stress both locally and systemically, thereby promoting neuroinflammation, neuronal death, cognitive decline, and reduced intestinal motility, as demonstrated in PD mouse models (Houser and Tansey 2017; Perez‐Pardo et al. 2019; Zhao et al. 2021).

In parallel with these inflammatory processes, several studies have reported a reduction in the abundance of the family *Prevotellaceae* in both fecal and colonic mucosal samples from PD patients, which is associated with decreased levels of mucin, short‐chain fatty acids (SCFAs), and neuroprotective factors (Keshavarzian et al. 2015; Scheperjans et al. 2015; Unger et al. 2016; Bedarf et al. 2017; Petrov et al. 2017; Lin et al. 2019; Zhang, Xu, et al. 2023). A marked decline in this bacterial family has also been correlated with reduced circulating levels of the hormone ghrelin during PD progression. Ghrelin influences mitochondrial respiration, modulates ROS levels, inhibits α‐synuclein accumulation, and regulates dopaminergic neuron function in the substantia nigra and striatum in PD animal models (Andrews et al. 2005; Conti et al. 2005; Andrews et al. 2009; Bayliss and Andrews 2013; Lin et al. 2019; Zhang, Ye, et al. 2023).

Among the most relevant microbial metabolites are the SCFAs, central mediators of gut–brain communication and immune regulation. In fecal samples from PD patients, their reduced availability has been consistently linked to the pronounced loss of SCFA‐producing bacteria, particularly members of the *Lachnospiraceae* family (Unger et al. 2016; Tan et al. 2021; Chen et al. 2022; Voigt et al. 2022; Yang, Ai, et al. 2022). Low levels of acetate, propionate, and butyrate can drive metabolic alterations, as the oxidation of these metabolites is estimated to account for approximately 8% of total daily energy expenditure in humans (Astbury and Corfe 2012; Blaak et al. 2020; Elford et al. 2024). Moreover, butyrate exerts a dual influence: it modulates the peripheral immune system, thereby indirectly affecting the CNS, and it can also act directly on the CNS, despite being largely metabolized in the periphery (Filiano et al. 2015; Pierre and Pellerin 2005; Kekuda et al. 2013). In the gut, butyrate additionally stimulates the secretion of serotonin, glucagon‐like peptide‐1 (GLP‐1), peptide YY (PYY), insulin, and amylin in the gastrointestinal tract. The release of these molecules can activate the vagus nerve and initiate neuroendocrine signaling pathways (Fukumoto et al. 2003; Cryan et al. 2019). There is strong evidence that reduced fecal butyrate levels have been associated with worsened symptoms, including postural instability, gait disturbances, cognitive impairment, motor dysfunction, and depressive symptoms (Tan et al. 2021; Chen et al. 2022; Wu et al. 2022). In vitro studies have demonstrated that sodium butyrate (NaB), for example, exerts neuroprotective effects against α‐synuclein aggregation (Chen et al. 2025; Getachew et al. 2020; Paiva et al. 2017; Sadeghloo et al. 2025; Zhang et al. 2022). However, Zhang and colleagues reported that acetate and propionate did not protect cells from rotenone‐induced toxicity. They attributed NaB's protective effects to its capacity to modulate autophagic responses to toxic α‐synuclein species via histone deacetylase (HDAC) inhibition, thereby reducing dopaminergic neurodegeneration (Zhang et al. 2022).

These findings underscore the potential therapeutic relevance of restoring gut microbiota homeostasis in PD treatment. Nevertheless, the current literature remains far from fully elucidating the mechanisms underlying this complex connection. Thus, rather than focusing solely on microbial community composition, the spectrum of their metabolic products represents the most promising avenue to understand and therapeutically target PD progression—a process in which the ENS emerges as a pivotal mediator of gut–brain communication.

## 4 ENS‐Microbiota Interactions and Their Implications for PD

**FIGURE 2:**
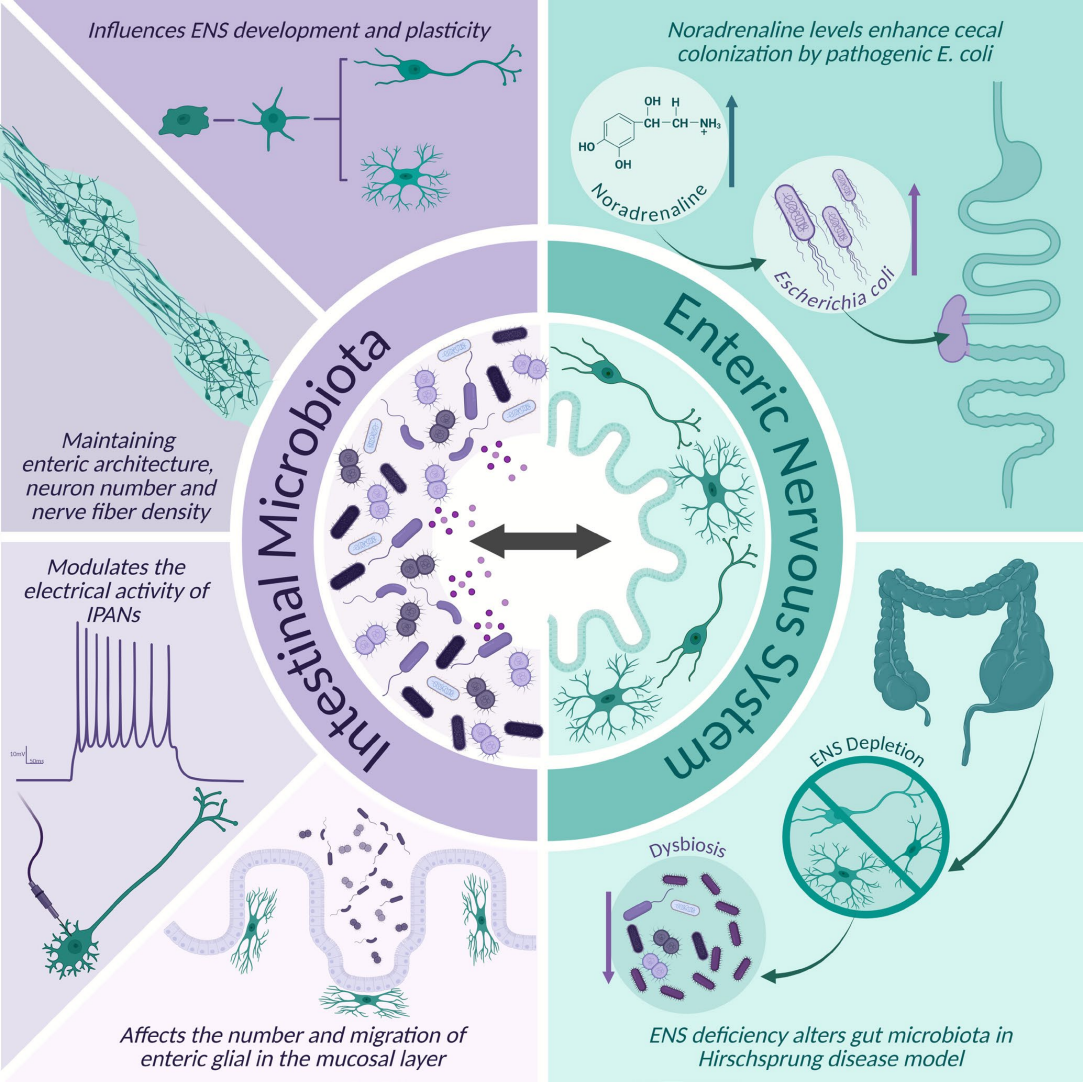
Bidirectional crosstalk between the intestinal microbiota and the enteric nervous system. The microbiota modulates enteric nervous system (ENS) development, architecture, neuronal activity, and number of enteric glial cells and migration, while ENS activity influences microbial composition, exemplified by noradrenaline‐driven *Escherichia coli* colonization. ENS depletion or dysfunction induces dysbiosis, disrupting microbiota–ENS homeostasis.

Given that both the ENS and gut microbiota are altered in PD, understanding how these two systems interact is critical for unraveling PD‐related gastrointestinal dysfunction. It has been extensively reported that the ENS responds to changes in the composition of the microbiota, as well as to its metabolites, including those altered in patients with PD. The crosstalk between the gut microbiota and the ENS occurs through both direct and indirect mechanisms; however, it remains unclear which specific ENS–microbiota signaling pathways are disrupted in PD and whether these alterations precede, accompany, or result from early α‐synuclein pathology in the gut. This section provides an overview of the main findings on ENS–microbiota interactions, discussing potential targets that may be relevant for PD pathology (Figure 2).

### 4.1 Bidirectional Regulation Between the ENS and the Gut Microbiota

Multiple studies in germ‐free (GF) rodents, which lack gut microbiota, have demonstrated that these animals exhibit an immature ENS, indicating that the microbiota profoundly influences ENS development and plasticity (Hyland and Cryan 2016). Evidence shows that the presence of gut microbiota is essential for maintaining normal myenteric plexus architecture and size, as well as nerve fiber density (Collins et al. 2014; Lomasney et al. 2014). Consistently, the number of myenteric neurons is decreased in the jejunum, ileum, and colon of GF mice (McVey Neufeld et al. 2015). Certain subpopulations, such as nitrergic and calbindinergic enteric neurons, appear to be more susceptible, as their numbers are significantly reduced in GF mice (Collins et al. 2014; McVey Neufeld et al. 2015). Intrinsic primary afferent neurons (IPANs), the main sensory neurons of the ENS, display altered electrophysiological properties in GF mice, characterized by decreased excitability (McVey Neufeld et al. 2013). Furthermore, the number of enteric glial cells in the mucosa of GF mice is reduced, which may contribute to impaired epithelial barrier integrity and function (Kabouridis et al. 2015; Rao and Gershon 2015). These findings establish a causal role for the microbiota in ENS maturation.

There is also strong evidence that the microbiota profile modulates ENS function. Abnormalities observed in the ENS of GF mice can be reversed by colonization with conventional microbiota (de Vadder et al. 2018; Heiss and Olofsson 2019). Specific bacterial strains have been shown to modulate the ENS, affecting its neurochemistry and neural activity (Kunze et al. 2009; Lomasney et al. 2014). Additionally, the gut microbiota appears to regulate the migration of enteric glia from the ENS to the mucosa, as demonstrated in antibiotic‐treated Sox10::Cre; R26RConfetti mice, where treatment reduced enteric glial staining in the mucosa (Kabouridis et al. 2015; Rao and Gershon 2015).

While the microbiota regulates enteric neural development and plasticity, ENS activity can, conversely, modulate gut microbiota diversity. For instance, increased levels of noradrenaline enhance the adherence of pathogenic *Escherichia coli* in the cecum of mice (Beata et al. 2021). Moreover, a mouse model of Hirschsprung disease, a congenital condition characterized by colorectal aganglionosis, intestinal inflammation, and constipation, showed marked differences in microbiota diversity, suggesting that the absence of ENS influences microbiota composition. Supporting this hypothesis, subsequent animal studies reported colonic bacterial overgrowth with an increased abundance of pro‐inflammatory strains (Chantakhow et al. 2021; Shi et al. 2024). Hirschsprung disease patients with associated enterocolitis also display dysbiosis, with significant beta‐diversity differences compared to healthy individuals (Shi et al. 2024). Taken together, these findings indicate that the ENS and gut microbiota dynamically and bidirectionally regulate each other's functions. Investigating this crosstalk offers promising insights into gastrointestinal and gut–brain axis physiology and dysfunction.

### 4.2 Mechanisms Underlying ENS–Microbiota Crosstalk

Although ENS–gut microbiota interactions are known to play a significant role in health and in both gastrointestinal and neurological disorders, the exact mechanisms underlying these interactions remain incompletely understood. How they occur in PD is still largely unknown. Nevertheless, some studies have investigated direct and indirect mechanisms involved in ENS–microbiota crosstalk in health and disease. Understanding these mechanisms may provide insights into how the ENS and gut microbiota interact in PD and potentially reveal novel therapeutic targets.

Obata et al. described a microbiota‐dependent neuronal programming circuit that regulates intestinal physiology. Their study demonstrated that the gut microbiota induces the expression of the aryl hydrocarbon receptor (AHR), a transcription factor expressed in neurons of the distal gastrointestinal tract that functions as a biosensor within intestinal neural circuits. This pathway enables neurons to respond to luminal signals and promote gut motility. Conversely, depletion of either AHR or the microbiota resulted in reduced peristaltic activity in mice, which could be restored by re‐expression of AHR in enteric neurons (Obata et al. 2020). Whether AHR‐dependent enteric circuits are dysregulated in PD remains unknown; such investigations could clarify whether impaired microbial sensing contributes to early ENS‐driven motility defects.

Serotonin is a critical neurotransmitter in the gastrointestinal tract, regulating motility, ENS development, and gut–brain communication (Mittal et al. 2017; Najjar et al. 2023). Approximately 95% of the body's serotonin is synthesized in the gut, where the microbiota plays an important role by stimulating enterochromaffin cells to produce serotonin (Gershon 2013; Legan et al. 2022). Yano et al. showed that GF mice have reduced serotonin production in the gut. This reduction was associated with decreased activation of serotonin 4 receptor (5‐HT4R)–expressing enteric neurons, leading to slowed gut motility (Yano et al. 2015). Subsequent studies demonstrated that gut microbiota regulates the maturation of the adult ENS through enteric serotonin networks (de Vadder et al. 2018). Emerging evidence indicates that certain gut microbes convert tryptophan into tryptamine, and the colonization of GF mice improved gastrointestinal transit by increasing mucosal serotonin production and stimulating the proliferation of enteric neuronal progenitors via 5‐HT4R activation (de Vadder et al. 2018; Fan et al. 2025). Although the role of enteric serotonin in PD is unclear, strong evidence suggests it is an important player in several gut–brain disorders (Generoso et al. 2021; Hung et al. 2025). Enteroendocrine cells, including enterochromaffin cells, express alpha‐synuclein and have been implicated in PD pathophysiology (Chandra et al. 2023). Studies have reported a significant reduction in 5‐HT levels across various regions of the brain, as well as in plasma and cerebrospinal fluid, in both patients and animal models of PD (Fan et al. 2025; Kamińska et al. 2017). Moreover, decreased serotonin levels correlate with the severity of depression in PD patients, indicating serotonergic system involvement (Khowdiary et al. 2025). Considering the microbiota's role in serotonin production and the importance of enteric serotonin in gastrointestinal and gut–brain physiology, future studies should investigate the mechanisms by which PD‐related dysbiosis affects enteric serotonergic circuits and related symptoms.

The ENS function can also be indirectly modulated by the gut microbiota through microbial metabolites such as SCFAs. SCFAs promote the release of GLP‐1 from enteroendocrine cells (Delzenne et al. 2005; Psichas et al. 2015). GLP‐1 activates gut–brain axis pathways that regulate food intake and, through receptors expressed in enteric neurons, induces nitric oxide release, leading to gastric antrum relaxation and inhibition of postprandial motility (Rotondo et al. 2011). Notably, PD patients exhibit abnormal gastric emptying and decreased microbiota‐derived SCFA production, although the mechanisms remain unclear (Chen et al. 2022; Safarpour et al. 2022).

SCFAs, particularly butyrate, have been increasingly linked to PD pathology and are critical for ENS maintenance. Wang et al. demonstrated that intestinal neurons proliferate more effectively in the presence of butyrate, while reduced butyrate levels impair ENS neuronal proliferation, worsening symptoms of functional constipation (Wang et al. 2023). Butyrate also prevented myenteric neuronal loss in a mouse model of inflammatory bowel disease (Caetano et al. 2023). This study also reported colocalization of G‐protein coupled receptor 41 (GPR41) with enteric neurons immunoreactive to neuronal nitric oxide synthase (nNOS) and choline acetyltransferase (ChAT), suggesting that SCFAs may exert a direct influence on the ENS via this receptor (Caetano et al. 2023). Additionally, butyrate was capable of rescuing intestinal dysmotility while reducing neuroinflammation by decreasing GFAP expression in enteric glial cells in an amyotrophic lateral sclerosis model (Zhang et al. 2021). PD is characterized by decreased SCFA levels, which have been associated with neuroinflammation and neurodegeneration, as previously described. Evidence, including increased GFAP levels in the gut reported by our group and others, supports the involvement of ENS dysfunction in these processes (Thomasi et al. 2023; Thomasi et al. 2022). Although SCFA depletion is consistently observed in PD patients, whether reduced SCFA signaling causally contributes to ENS dysfunction remains unresolved. Both enteric neurons and glial cells express Toll‐like receptors (TLRs), which recognize microbial components such as LPS and serve as important mediators of microbiota–host communication (Barajon et al. 2009). Evidence suggests that LPS affects enteric neuronal survival in a concentration‐dependent manner and modulates myenteric neuron activity by inducing nitric oxide production (Anitha et al. 2012; Voss and Ekblad 2014). TLRs have been implicated in regulating ENS neurochemical profiles, as well as neuronal and glial survival and activity. Interestingly, mice lacking TLR4 expression exhibit ENS neurochemical profiles similar to those of GF and antibiotic‐treated mice, including reduced nitrergic neurons (Anitha et al. 2012). Moreover, antibiotic‐treated wild‐type mice develop ENS abnormalities that can be reversed by supplementation with a TLR2 agonist, supporting the role of TLR signaling as a key communication pathway between the ENS and gut microbiota (Brun et al. 2013).

TLRs are also critical for enteric glial function. TLR2 regulates enteric glia–derived production of glial cell line–derived neurotrophic factor (GDNF), an essential factor mediating glial effects on intestinal epithelial barrier integrity (Brun et al. 2013). A study using human enteric glial cells provided a direct link between microbiota–enteric glia crosstalk and TLR signaling: stimulation of primary human enteric glia cultures with pathogenic bacteria or probiotics differentially modulated TLR expression, resulting in distinct S100 calcium‐binding protein B (S100B) expression and nitric oxide release (Turco et al. 2014). Similarly, Yang et al. showed that specific bacterial strains modulate enteric glial expression of neuroinflammation‐related factors such as TLR2, major histocompatibility complex II, GDNF, and tumor necrosis factor alpha (TNF‐α) (Yang, Li, and Le 2022). These findings suggest that the microbiota interacts with enteric glial cells to regulate intestinal inflammatory responses. Gut neuroinflammation is a key process in PD pathology, where enteric glial cells play a central role. Compelling evidence indicates that enteric glia in PD are activated and display altered expression of GFAP, GDNF, S100B, and pro‐inflammatory cytokines, including TNF‐α (Thomasi et al. 2024; Thomasi et al. 2022). Furthermore, TLR2 and TLR4 have also been implicated in PD pathogenesis (Gorecki et al. 2021). However, it remains unclear whether PD‐associated dysbiosis drives enteric glial dysfunction, or vice versa, despite indirect evidence suggesting such an effect.

In summary, current literature indicates that the ENS and gut microbiota communicate through multiple direct and indirect pathways in both health and disease. These pathways are essential for ENS development and plasticity, gastrointestinal physiology, and gut–brain interactions. Furthermore, ENS–microbiota crosstalk contributes to gastrointestinal dysfunction by influencing gut neurochemistry, neuronal activation, and neuroinflammatory responses. The evidence reviewed here provides a foundation for future investigations and represents potential entry points for therapeutic intervention. However, it remains elusive which of these pathways are indeed altered and whether they cause or are secondary to neurodegeneration in PD.

### 4.3 ENS–Gut Microbiota Interactions Potential Relationship With PD Pathology Spread via the Vagus Nerve

Both the ENS and the gut microbiota are involved in the underlying pathological mechanisms of PD, and their interactions potentially contribute to gastrointestinal dysfunction. Notably, evidence also suggests that both are implicated not only in gut dysfunction but also in the spread of pathology from the gut to the CNS via the vagus nerve, the main anatomical connection between the two organs. The vagus nerve has been shown to play a central bidirectional role in PD, with both afferent and efferent signaling involved in pathological mechanisms. Direct transmission of α‐synuclein from enteric neurons to the brain through vagal afferents has been extensively demonstrated in different PD animal models, and epidemiological studies have found that vagotomy is associated with decreased PD incidence (Holmqvist et al. 2014; Kim et al. 2019; Svensson et al. 2015).

In addition to mediating pathology transmission, vagal function is also impaired in PD. Patients exhibit dysautonomia, which is a dysregulation of the autonomic nervous system and includes cardiovascular dysfunction, orthostatic hypotension, urinary problems, and gastrointestinal dysmotility (Chen et al. 2020; Huckemann et al. 2022). Moreover, the vagus nerve is a major regulator of immune homeostasis, which is disrupted in PD patients, as neuroinflammation is a key pathological process of the disease (Pavlov and Tracey 2005). Consistently, the vagal cholinergic anti‐inflammatory signaling appears to be reduced in 6‐hydroxydopamine (6‐OHDA) PD rats, in which a decreased number of cholinergic neurons in the dorsal motor nucleus of the vagus (DMV) is associated with gastric inflammation and gastroparesis (Zheng et al. 2011). Interestingly, activation of gastric α7AChR, the receptor engaged by the vagal anti‐inflammatory cholinergic pathway, reverses inflammation and dysmotility (Zhou et al. 2021).

It is possible that both the ENS and the gut microbiota contribute to the disruption of vagal anti‐inflammatory function in PD. Vagal afferent and efferent fibers communicate closely with enteric neuron subtypes. For example, IPANs form synapses with vagal fibers, conveying sensory information from the gut to the brain, and although this connection has not been investigated in PD research, it has been shown to be impaired during aging (McVey Neufeld et al. 2024). The vagus nerve also interacts with enteric glial cells. Evidence from preclinical studies indicates that, under physiological conditions, enteric glia can mediate the anti‐inflammatory effects of vagal signaling on resident intestinal immune cells following intestinal injury (Bonaz 2022; Langness et al. 2017). Conversely, vagotomy prevents the increase in GFAP expression—indicative of enteric glial activation—in the intestine in a mouse model of intestinal ischemia (Costantini et al. 2010). In PD, enteric glia are found in a reactive state in both patients and animal models, promoting gut neuroinflammation. However, whether this contributes to vagal dysfunction remains unexplored, although it would be plausible.

Studies have also shown that the microbiota can influence vagal signaling properties (Cawthon and De La Serre 2018; Li et al. 2018). A recent study found that transplanting healthy mice with fecal material from PD patients induced gut–brain PD pathology, including gut neuroinflammation and α‐synuclein accumulation in the DMV, suggesting that PD‐related dysbiosis drives symptoms through the transmission of pathology from the gut to the brain via the vagus nerve (Sampson et al. 2016). Indeed, vagal afferents respond to microbiota‐derived metabolites. For example, SCFA receptors such as GPR41 are expressed in vagal terminals in the gut, and their activation by butyrate triggers action potentials (Lal et al. 2001). Therefore, it is possible that reduced SCFA levels decrease vagal tone in the gut, although no direct evidence currently supports this. Moreover, TLR4 is expressed on vagal afferent fibers, enabling them to sense bacterial products such as LPS (Perez‐Pardo et al. 2019). As discussed above, dysbiosis combined with breakdown of the intestinal epithelial barrier in PD patients results in increased LPS levels, which could also negatively affect vagal signaling (Bhattacharyya and Bhunia 2021). Additionally, several microbial neuroactive molecules are proposed to interact with vagal afferents in the gut and affect the CNS, and thus could contribute to PD‐related vagal dysfunction, given that dysbiosis is observed in patients. However, mechanistic studies are needed to elucidate how gut microbiota metabolites signal to and disrupt vagal function in PD.

Collectively, the ENS, the microbiota, and the vagus nerve appear to be interconnected and central to the complex pathological environment in the gut, influencing gastrointestinal function and PD progression to the brain. Dysbiosis leads to gut inflammation by increasing proinflammatory factors such as LPS and decreasing anti‐inflammatory factors such as SCFAs (Santos et al. 2025). Enteric glial cell activation is central to gut neuroinflammation, promoting cytokine release and disrupting epithelial barrier integrity (Thomasi et al. 2023). The anti‐inflammatory vagal role appears to be hijacked in the disease, while α‐synuclein pathology is transmitted to the CNS through vagal afferents. The evidence reviewed here provides a foundation for future investigations into the involvement of these interactions in PD‐related gastrointestinal and gut–brain dysfunction.

## 5 The Interactions Between the ENS and the Microbiome as a Potential Target for Management of PD Pathology

Given the evidence linking the ENS and gut microbiota in PD, as highlighted in this review, this section addresses nutritional strategies and microbiota‐targeted therapies as potential approaches to modulate this interaction. By focusing on the gut–brain axis, these interventions aim not only to alleviate gastrointestinal and neurological symptoms but also to influence underlying disease mechanisms, offering promising avenues for managing PD pathology.

### 5.1 Nutritional Strategies

PD has been the focus of various dietary interventions, including protein‐restricted diets, the ketogenic diet, the Mediterranean diet, and the Mediterranean‐DASH Intervention for Neurodegenerative Delay diet (Almeida et al. 2022). These dietary patterns have been investigated for their effects on PD risk, progression, and severity. However, studies specifically evaluating their impact on the ENS and gut microbiota remain limited, underscoring the need for further experimental data. This is particularly important, as dietary patterns can profoundly influence both the composition and function of the gut microbiota as well as ENS signaling (Almeida et al. 2022).

Within the CNS, caffeic acid has been shown to reduce dopaminergic neuronal loss in A53T α‐synuclein transgenic mice (Zhang et al. 2019). In a rotenone‐induced PD model, the same compound modulated enteric neurons, preventing neurodegeneration in the myenteric plexus, although no effects were observed in enteric glial cells (Zhang et al. 2019). These effects were attributed to the antioxidant properties of caffeic acid; however, this compound also modulates the gut microbiota in experimental models of intestinal disorders such as colitis, intestinal inflammation, and obesity (Wan et al. 2021; Wen et al. 2023; Fang et al. 2024). Notably, caffeic acid is present in coffee, and caffeine consumption has been associated with a reduced incidence of PD (Costa et al. 2010). Taken together, these findings suggest that caffeic acid may exert neuroprotective effects in PD through both direct actions on the CNS and ENS and indirect modulation of the gut microbiota.

Other compounds have also been associated with the alleviation of enteric neurodegeneration. Administration of docosahexaenoic acid (DHA), an omega‐3 polyunsaturated fatty acid, increased dopaminergic neuron density in the myenteric plexus and enhanced tyrosine hydroxylase (TH) + varicosities in Thy1‐αSyn transgenic mice (Lamontagne‐Proulx et al. 2021). Although the gut microbiota profile was not evaluated in this study, DHA has been shown to modulate the gut microbiota in other contexts, suggesting a potential mechanism through which it may influence the ENS (Zhang et al. 2020; Hosomi et al. 2021). Fish oil, a major dietary source of DHA and a component of the Mediterranean diet, has been associated with slower PD progression in clinical studies (Mischley et al. 2017). More recently, piperine was shown to ameliorate gastrointestinal dysfunction in a 6‐OHDA‐induced PD model, improving fecal water content, increasing SCFA levels, modulating gut microbiota composition, and reducing α‐synuclein accumulation in the colon (Yu et al. 2024). Additionally, polyphenols such as curcumin and resveratrol have demonstrated neuroprotective effects on the ENS and are recognized for their ability to modulate the gut microbiota, making them promising therapeutic strategies in PD (Yu et al. 2015; Ferreira et al. 2018; Ruan et al. 2022; D'Antongiovanni et al. 2023; Prakash et al. 2024).

### 5.2 Gut Microbiota‐Targeted Therapies

Probiotics have gained increasing attention in neurodegenerative diseases, and a recent systematic review concluded that they can improve constipation in PD patients (Jin et al. 2024). Whether this improvement is related to ENS–microbiota signaling remains unclear. Some probiotic strains have been investigated for their ability to modulate ENS activity. *Lactobacillus reuteri* and *Bifidobacterium longum* influence the electrophysiological properties of enteric neurons, enhancing motility and intestinal propulsion—an effect potentially relevant to PD (Wang et al. 2010; Khoshdel et al. 2013). *Saccharomyces boulardii* improved enteric neurodegeneration and inflammation in an experimental model of inflammatory bowel disease, leading to enhanced motility (Brun et al. 2017). *Lactobacillus rhamnosus* GG modulated redox responses in enteric neurons and improved stool frequency, total transit time, and ileal contractility (Chandrasekharan et al. 2019). *Bifidobacterium bifidum* CCFM1163 increased fecal water content and SCFA levels, improved gut microbiota composition, and reduced GFAP mRNA expression in a mouse model of induced constipation (Tang et al. 2023). Collectively, these findings highlight probiotics as a promising strategy for modulating the ENS and gut microbiota in PD.

Prebiotics have also emerged as promising candidates in PD. Trehalose has recently been identified as a potential modulator of both the ENS and gut microbiota. In a PD transgenic mouse model (PrP‐A53T G2‐3), 6 months of trehalose supplementation decreased TH immunoreactivity in the substantia nigra, striatum, and colon while increasing bacterial diversity in the gut microbiota (Pradeloux et al. 2024). Inulin, another prebiotic, has been explored for its effects on the ENS but has yielded inconsistent outcomes across models. In rodent models of diabetes and high‐fat diet, inulin supplementation did not significantly alter glial markers (GFAP, GDNF) in the colon or brain, nor did it affect myenteric neuronal subpopulations or enteric glial cells, despite reducing inflammation and improving colonic motility (Hosseinifard et al. 2019; Beraldi et al. 2020). More recently, however, inulin prevented gut dysbiosis in a spinal cord injury model, improving dysmotility and promoting enteric neuronal survival (Hamilton et al. 2024). Given the distinct gastrointestinal pathophysiology in PD, both trehalose and inulin warrant further investigation.

Within the scope of microbiota‐based therapies, SCFAs such as butyrate, propionate, and acetate have also emerged as relevant interventions capable of modulating the ENS. These molecules enhance Ca^2+^ responses in neurons and regulate myenteric neuron function (Fung et al. 2021). As noted above, butyrate in particular increases the number of nitrergic myenteric neurons both in vivo and in vitro and enhances contractile responses ex vivo (Soret et al. 2010). Furthermore, *Fusobacterium varium* , a butyrate‐producing bacterium, has been positively correlated with defecation frequency, and butyrate was shown to induce enteric neuron proliferation in vitro, suggesting a direct role in ENS‐mediated motility (Wang et al. 2023). In a model of experimental colitis, butyrate also prevented neuronal loss (Caetano et al. 2023). Collectively, these findings highlight the potential of SCFAs—particularly butyrate—as modulators of ENS function with protective effects against enteric neurodegeneration.

Fecal microbiota transplantation (FMT), though still experimental in neurodegenerative diseases, has yielded promising preliminary results in PD. By replacing a dysbiotic gut microbiota with that of a healthy donor, FMT may restore the gut ecosystem and reestablish proper gut–brain communication. In PD animal models, FMT modulated microbiota composition and intestinal permeability, prevented dopaminergic neuronal loss, inhibited α‐synuclein aggregation in the CNS, and restored striatal neurotransmitter levels, thereby improving motor symptoms (Sun et al. 2018; Zhao et al. 2021). In a clinical study, patients with mild to severe PD who received FMT once a week for three consecutive weeks showed improvements in PD symptoms and gastrointestinal outcomes, along with increased microbiota diversity, without adverse effects (Chen et al. 2022; Loh et al. 2024; Yadav and Raj 2025). Despite these encouraging results, neither clinical nor preclinical studies have specifically investigated the effects of FMT on ENS–microbiota interactions or their underlying signaling mechanisms. Additionally, potential adverse effects, such as immune reactions or pathogen transmission, remain concerns for this therapy and should be addressed in future studies.

In this context, targeting the gut microbiota and ENS through nutritional and microbiome‐based interventions represents a promising, non‐invasive approach to PD management. While preliminary evidence suggests therapeutic potential, the field remains underexplored, and further studies are needed to determine the most effective intervention types, combinations, and durations. Importantly, most existing studies have not simultaneously assessed the ENS and gut microbiota, limiting our understanding of their dynamic interplay in PD. A personalized approach that considers each patient's microbiota profile, disease stage, and comorbidities may ultimately provide the greatest therapeutic benefit from dietary and microbiota‐targeted strategies in PD.

## 6 Neglecting the ENS: A Critical Barrier to Understanding Gut‐Brain Axis Dysfunction in PD

Both the ENS and the gut microbiota are key players in gut–brain axis homeostasis and PD pathology. Extensive research shows that both contribute to disease initiation and progression through processes such as neuroinflammation and gut‐to‐brain α‐synuclein transfer, thereby contributing to gastrointestinal and neurological dysfunction. Although research on microbiota alterations in PD is abundant, it is increasingly evident that attempting to define a PD‐specific microbiota signature and develop microbiota‐based therapies presents substantial challenges.

First, microbiota composition is highly variable across populations, ages, sexes, lifestyles, and environments, making it extremely difficult to develop standardized treatments (Fenn et al. 2023; Shanahan et al. 2023; Sun et al. 2024). One potential solution is to focus on personalized therapies; however, the high costs associated with such approaches would likely limit their accessibility to PD patients worldwide. Second, our understanding of the gut microbiota as a whole remains incomplete. The optimal method for sampling microbiota for profiling is still debated, as stool samples often fail to detect individual‐level differences in the intestinal mucosal microbiota (Tang et al. 2020; Szóstak et al. 2022). Moreover, there is no consensus on what constitutes a “healthy” microbiota. Recently, Hul et al. argued that the concepts of a healthy or imbalanced microbiota should be linked to gut health. They emphasized the need for continued research and a nuanced approach to understanding this complex and evolving concept, highlighting the importance of more precise and inclusive definitions and methodologies in microbiota research (van Hul et al. 2024).

Given the challenges in profiling the microbiota and the incomplete understanding of its interactions with the host, microbiota modulation can lead to severe and unpredictable adverse effects. Indeed, mild to severe side effects have been reported in up to 39.3% of FMT procedures (Sahle et al. 2024). While most adverse effects are minor and transient, severe complications include transmission of multidrug‐resistant *Escherichia coli* , transmission of other infectious agents, and hospitalization (Sahle et al. 2024). Overall, researchers face substantial technical and logistical barriers to implementing safe, effective, and well‐regulated microbiota‐based treatments in clinical practice.

The magnitude of these challenges raises the question of whether microbiota modulation is a feasible approach for developing effective, precise, and safe PD therapies. While it is clear that the microbiota is essential for maintaining systemic homeostasis and that dysbiosis plays an important role in PD pathophysiology, concentrating research efforts exclusively on microbiota modulation may be an unproductive path.

Alternatively, targeting the signaling pathways between the microbiota and the host may offer an opportunity to harness the therapeutic potential of the microbiota while enabling the development of more precise and effective treatments. Although microbiota–host crosstalk is highly complex, a deeper understanding of these interactions is essential for this strategy. In this context, the ENS is a central player in gut–brain communication and PD pathology that has been largely overlooked in microbiota research. In this review, we have outlined crosstalk pathways between the ENS and the microbiota, based on current evidence, that are relevant for understanding and treating gut–brain dysfunction in PD. Investigating how the ENS and gut microbiota interact, both directly and indirectly, and how this interaction impacts PD‐related gastrointestinal and gut–brain dysfunction represents an urgent and promising research avenue that could yield critical insights and inform the development of novel therapeutic targets.

Taken together, these considerations point to the need for a conceptual shift in how gut involvement in PD is approached. Rather than seeking a definitive microbial profile, future work should focus on functional outputs, such as microbial metabolites, that more directly impact host physiology and can serve as clinically relevant biomarkers. Yet, identifying these molecules is only the first step. The critical challenge ahead lies in deciphering how the enteric nervous system perceives, integrates, and responds to such signals. By focusing on this neuro–metabolic interface, future research may move beyond descriptive correlations to mechanistic understanding, clarifying the role of the microbiota within the gut–brain axis. Importantly, strategies that directly modulate enteric neuronal and glial populations, or that experimentally probe how these cell types respond to specific microbial metabolites in PD models, may provide a means to determine how ENS–microbiota communication can influence early gut dysfunction and downstream brain pathology. Ultimately, this perspective positions the ENS not as a passive target, but as an active mediator whose interactions with microbial metabolites may reveal novel therapeutic avenues and refine strategies for managing both gastrointestinal and neurological manifestations of PD. Although evidence from other gut–brain axis disorders remains limited, this largely reflects the early stage of ENS‐focused research rather than a lack of biological relevance. A clearer understanding of the ENS is therefore needed to determine its potential role as an early interface through which gut‐derived signals influence brain function.

## Author Contributions

**Luisa Valdetaro:** writing – original draft, data curation. **Maria Carolina Ricciardi:** writing – original draft, visualization, data curation. **Patricia Pereira Almeida:** writing – original draft, data curation. **Milena Barcza Stockler‐Pinto:** writing – review and editing. **Ana Lucia Tavares‐Gomes:** conceptualization, writing – review and editing, supervision.

## Funding

This work was supported by Conselho Nacional de Desenvolvimento Científico e Tecnológico, Process number 313320/2025‐0. Fundação Carlos Chagas Filho de Amparo à Pesquisa do Estado do Rio de Janeiro, Process number E‐26/210.126/2025. Coordenação de Aperfeiçoamento de Pessoal de Nível Superior, Finance Code 001.

## Conflicts of Interest

The authors declare no conflicts of interest.

## Data Availability

Data sharing is not applicable to this article because no new data were created or analyzed in this study.
